# Platelet-Rich Plasma for Bone Fracture Treatment: A Systematic Review of Current Evidence in Preclinical and Clinical Studies

**DOI:** 10.3389/fmed.2021.676033

**Published:** 2021-08-03

**Authors:** Yangming Zhang, Fei Xing, Rong Luo, Xin Duan

**Affiliations:** Department of Orthopaedics, West China Hospital, Sichuan University, Chengdu, China

**Keywords:** platelet-rich plasma, platelet, fracture, osteoblasts, growth factors, systematic review

## Abstract

**Background:** Recently, there is an increasing interest in the therapeutic potential of platelet-rich plasma (PRP) for bone fracture treatment. Nevertheless, the effect of PRP for bone fracture treatment remains controversial and is still a matter of discussion. Therefore, we performed a systematic review to evaluate the efficacy and safety of PRP injection for treatment of bone fracture.

**Methods:** The main bibliographic databases, including Medline, PubMed, Embase, Web of Science, and the Cochrane library, were comprehensively searched for studies focusing on the application of platelet-rich plasma (PRP) on bone fracture treatment. All relevant articles were screened for eligibility and subdivided into the preclinical and clinical studies. Data were extracted and presented systematically.

**Results:** Finally, twenty-six *in vitro* preclinical studies (basic studies), nine *in vivo* preclinical studies (animal studies), and nine clinical studies, met the selection criteria, and were included in the present systematic review. Preclinical studies showed an overall positive effect of PRP on osteoblast-like cells *in vitro* and bone healing in animal models. The most used treatment for bone fracture in animal and clinical studies is fixation surgery combined with PRP injection. The clinical studies reported PRP shortened bony healing duration, and had no positive effect on improving the healing rate of closed fractures. However, the results of functional outcomes are controversial. Additionally, compared with control group, PRP would not increase the rate of postoperative wound infection.

**Conclusion:** The present systematic review confirmed the continuing interests of PRP as an additional treatment for bone fracture. Preclinical studies highlighted the potential value of PRP as promising therapy for bone fracture. However, the preclinical evidence did not translate into a similar result in the clinical studies. In addition, types of fractures and procedures of PRP preparation are heterogeneous in enrolled studies, which might result in controversial results. Meanwhile, characteristics of PRP, such as platelet concentration, the numbers of leukocytes, still need to be determined and further research is required.

## Introduction

Bone tissue is a major part of the musculoskeletal system and provides the framework which supports the body and maintains its shape (1–3). Although bone tissue has the potential for spontaneous healing after injuries, the regenerative capacity of bone tissue is limited by many factors, such as age, type of fracture, genetic bone disorder (4–6). Additionally, up to 13% of tibial shaft fractures are associated with fracture non-union or delayed union, which are the most devastating complications of traumatic fractures (7–9). For elderly patients with lower limb fracture, the long period of bedridden time and immobilization increases the incidence of pulmonary infection, thrombosis, and bedsore, and as a consequence the risk of death (10, 11). Currently, the principle of clinical treatment for a fracture is reduction and fixation. Meanwhile, many clinical approaches, such as administration of bone morphogenetic proteins (12, 13), cell-based therapies (14, 15), platelet-rich plasma (16), or implantation of graft biomaterials (17), have been used either alone or in combination to enhance bone regeneration. Additionally, many previous studies demonstrated that low-intensity pulsed ultrasound (LIPUS) could also accelerate fracture healing and increase the rate of fracture healing (18, 19).

Deriving from centrifugation of peripheral blood, PRP can deliver a high concentration of autologous bioactive factors, including transforming growth factor-beta, platelet-derived growth factor, and interleukin, in a low cost and minimally invasive way (20). The bioactive factors released from PRP can take part in the process of neovascularization, tissue remodeling, and regulation of inflammation, which led to the idea of using PRP for tissue repair (21, 22). After further freeze-thawing and centrifugation steps, resulting in the lysis of platelets, PRP can turn into platelet lysate, which contains higher concentrations of growth factors (23). Additionally, PRP can combine with thrombin and calcium to form a coagulum, called platelet gel (24). Platelet-poor plasma is the residual plasma once the PRP is extracted, which still contains beneficial proteins, insulin-GF and a low number of platelets (25). In the last decade, as a fashionable treatment, PRP has shown sustained beneficial repair effects in clinical procedures involving various soft tissue, such as ligaments and tendons (20). Meanwhile, there is an increasing interest in the therapeutic potential of PRP for bone fracture treatment. Nevertheless, the effect of PRP for bone fracture treatment remains controversial and is still a matter of discussion. As there is no related systematic review published yet, we performed this systematic review to evaluate whether PRP injection improve outcome of bone fracture, in terms of bony union time, bone healing rate, functional scores, VSA scores, complication, and imaging.

## Methods

### Search Strategy

This systematic review was performed according to the Preferred Reporting Items for Systematic reviews and Meta-Analysis (PRISMA) guidelines (26). Two reviewers independently searched for potentially relevant published researches using electronic databases, including Medline, PubMed, Embase, Web of Science, and the Cochrane library from inception to March 2020. The search strategy for all electronic databases was built as follows: “(PRP OR platelet-rich plasma OR plasma rich in growth factors OR platelet derived growth factor OR autologous plasma OR autologous conditioned plasma) and (bone fracture OR fracture OR fractures OR osteoblast-like cells OR osteoblasts)”. The electronic database search was supplemented by a manual search of the reference lists of included articles. The titles and abstracts of the search results were initially evaluated, and then the full-text manuscript was reviewed. Disagreements were resolved by discussion, and a third author conducted an independent review if the agreement was not reached.

### Eligibility Criteria

The inclusion criteria for all studies were as follows: (1) Original research. (2) Studies reporting the procedure of PRP preparation. (3) Studies written in English. Additional inclusion criteria for *in vitro* basic studies were as follows: (1) The domain had to be an osteoblast-like cell line. (2). Studies reporting the *in vitro* effect of PRP on osteoblast-like cells. Additional inclusion criteria for *in vivo* preclinical studies (animal studies) and clinical studies were as follows: (1) Prospective or retrospective controlled studies. (2) Studies involving the local application of PRP for bone fracture treatment. The exclusion criteria for all studies were as follows: (1) Duplicated publications. (2) Case reports, comment papers, and correspondence. (3) Reviews. (4) Studies involving stem cells or other biomaterial scaffolds. Furthermore, any disagreements were resolved by discussion and consensus with a third reviewer.

### Data Extraction and Analysis

Two reviewers independently extracted data from each included study. The following data were extracted from *in vitro* preclinical studies: author, center, types of cells, effects of PRP on cells. The following data were extracted from *in vivo* preclinical studies: author, medical center, animal model, types of bone, treatment groups, PRP injection volume, the period of follow up, the procedure of PRP preparation, outcomes. The following data were extracted from clinical studies: author, medical center, study design, the number of patients, the average age of participants, types of bone, PRP injection volume, the period of follow-up, the procedure of PRP preparation, clinical outcomes. Due to the high heterogeneity of the included studies, a quantitative evaluation of the results was not performed. If there was a dispute between the two reviewers, it was settled through consultation with a third reviewer.

### Assessment of Methodological Quality

Two reviewers independently evaluated the methodological quality of studies in this systematic review, according to the criteria in the Cochrane Collaboration for Systematic Reviews (27). The quality of the *in vivo* preclinical studies in this systematic review was assessed using the items of the Systematic Review Centre for Laboratory animal Experimentation (SYRCLE) risk of bias (RoB) tool (28). The quality of the clinical studies in this systematic review was assessed using the Cochrane RoB tool (29). Furthermore, any disagreements were resolved by discussion and consensus with a third reviewer.

## Results

**Figure 1:**
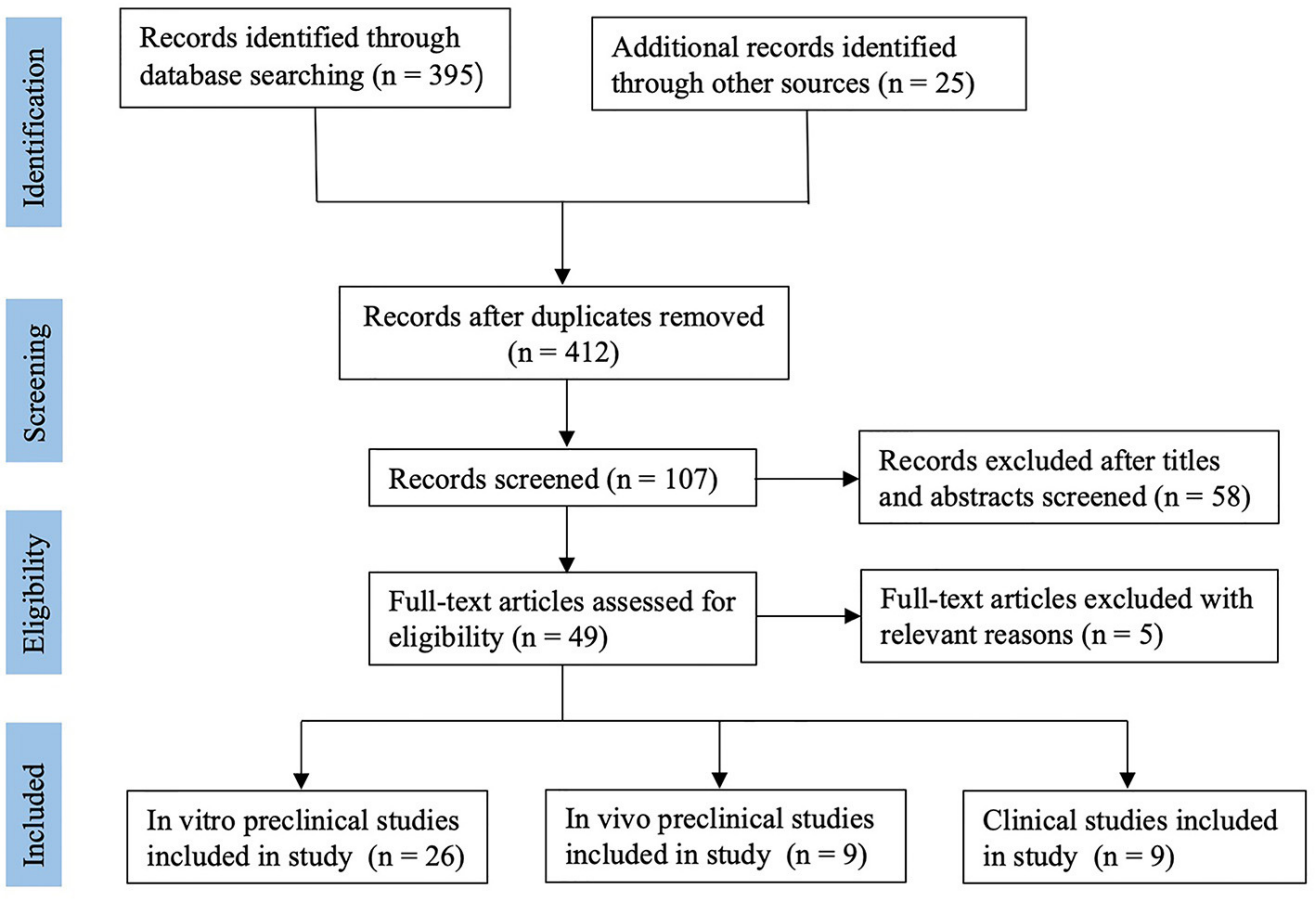
Flow chart of study selection.

**Figure 2:**
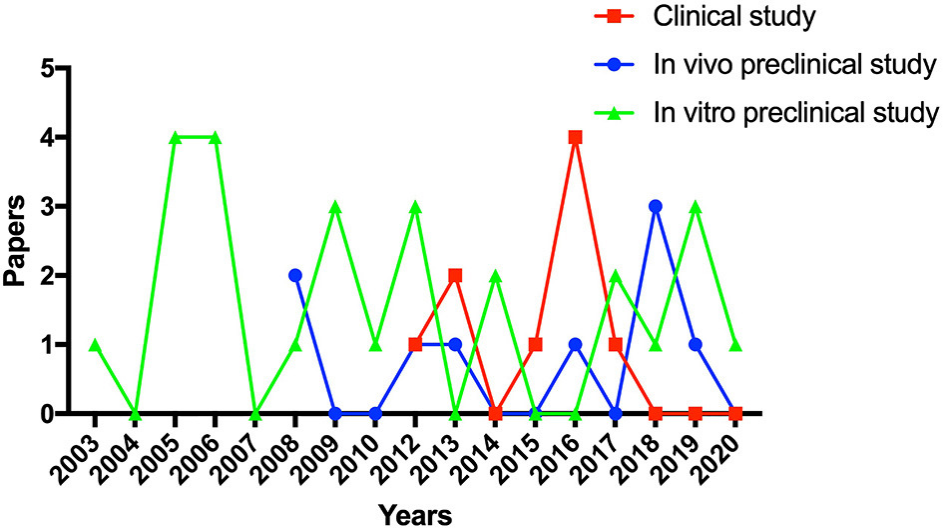
The trend of preclinical and clinical studies published over time.

According to the search strategy, 412 relevant publications were screened. Finally, forty-four studies, including twenty-six *in vitro* preclinical studies (basic studies), nine *in vivo* preclinical studies (animal studies), and nine clinical studies, met the selection criteria and were enrolled in the present systematic review. The flow diagram of this systematic review is shown in Figure 1. The trend of preclinical and clinical studies published over time is reported in Figure 2. Details of all enrolled studies are summarized in the present systematic review, and the main results will be discussed separately for preclinical and clinical studies in this systematic review.

### Preclinical Studies

#### In Vitro Preclinical Studies

Twenty-six studies (22, 30–54) investigated the effect of PRP on osteoblast-like cells. The main details of *in vitro* studies are shown in Table 1. Among these studies, six studies were performed in Italy, six in Japan, two in Germany, two in Iran, two in China, two in Brazil, each one in Australia, Spain, USA, Netherlands, South Korea, and Czech Republic. Many kinds of osteoblast-like cells, including osteoblasts, MG-63 cells, SaOS-2 cells, MC3T3-E1 cells, were discussed in the present systematic review. Seventeen out of all enrolled *in vitro* preclinical studies reported that PRP enhanced the proliferation of osteoblast-like cells. Seven out of all enrolled studies focused on the effect of PRP on the differentiation, including six studies with positive outcomes, and one study with negative outcomes. Among all enrolled *in vitro* preclinical studies, four studies found that PRP improved the cell viability of osteoblast-like cells, four with positive outcomes in stimulating the migration of osteoblast-like cells, two with positive outcomes in enhancing the adhesion of osteoblast-like cells. Additionally, one study reported PRP application in osteoblast cultures leads to higher levels of platelet-derived growth factor (PDGF), insulin-like growth factor I (IGF1), and transforming growth factor (TGF) release than platelet-rich fibrin (44). One study reported that PRP application in osteoblast cultures leads to higher levels of TGF, PDGF, vascular endothelial growth factor (VEGF), hepatocyte Growth Factor (HGF), epidermal growth factor (EGF), and IGF1 release than cell medium with 5% or 15% fetal bovine serum (FBS) (22). Two out of all enrolled studies reported growth factor profile of PRP. Ogino et al. reported that the mean levels of TGF-β1, PDGF-AB, and IGF-I in PRP prepared by double-centrifugation were 0.190 ± 0.039, 0.271 6 ± 0.043, and 0.110 ± 0.039 ng/1500 × 10^3^ platelets, respectively (49). Okuda et al. also found that the levels of PDGF-AB, and TGF-β1 were also concentrated up to 182.0 ng/ml and 140.9 ng/ml, respectively (54). Only two studies focused on mechanisms of the PRP effect. Casati et al. demonstrated that PDGF contained in PRP stimulates migration of osteoblasts by reorganizing actin cytoskeleton (37), while Kinoshita et al. found that PRP induced osteoblast proliferation via PDGF receptor-mediated signal transduction (30).

**Table 1:** The main details of *in vitro* preclinical studies.

**Author (Year)**	**Center**	**Types of cells**	**Effect of PRP on cells**
Kinoshita et al. (2020) (30)	Chiba, Japan	Human osteoblasts	Both fresh-PRP and freeze-dried-PRP significantly induced osteoblast proliferation.
Fernández-Medina et al. (2019) (31)	Herston, Australia	Human osteoblasts	Cell viability and migration assay have demonstrated a detrimental effect when the concentration was>60%.
Steller et al. (2019) (32)	Luebeck, Germany	Osteoblasts	The negative effect of zoledronic acid on cell proliferation was especially reduced by PRP and platelet-rich fibrin (PRF).
Vahabi et al. (2019) (33)	Tehran, Iran	MG-63 cells	Activated PRP had a positive effect on the viability and adhesion of osteoblast-like cells.
Wang et al. (2018) (34)	Wuhan, China	Human osteoblasts	PRP enhanced cell adhesion, proliferation, osteoblast differentiation.
Kobayashi et al. (2017) (35)	Niigata, Japan	Human osteoblast	PRP tended to have little to no effect on osteoblast differentiation.
Vahabi et al. (2017) (36)	Tehran, Iran	MG-63 cells	The current study failed to show the significant effect of activated or non-activated PRP on proliferation of MG-63 osteoblast-like cells.
Casati et al. (2014) (37)	Milano, Italy	Human osteoblast–derived osteosarcoma (SaOS-2) cells	Platelet derived growth factor contained in PRP stimulates migration of osteoblasts by reorganizing actin cytoskeleton.
Martinotti et al. (2014)	Alessandria, Italy	SaOS-2 cells	PRP induces the development of mixed osteogenic/osteoclastogenic traits in the SaOS-2 cells.
Herrera et al. (2012) (39)	Araraquara, Brazil	SaOS-2 cells	PRP can stimulate osteoblast activity and cytokine/chemokine release.
Mazzocca et al. (2012) (22)	Farmington, USA	Human osteoblasts	PRP significantly increased the proliferation of osteoblasts.
Garcia-Martinez et al. (2012) (40)	Granada, Spain	Human osteoblasts	PRP increased the proliferation of human osteoblasts.
Mooren et al. (2010) (41)	Nijmegen, Netherlands	Rat osteoblasts	The proliferation of osteoblast-like cells can significantly be enhanced by supplementation of PRP derivatives.
Colciago et al. (2009) (42)	Milano, Italy	SaOS-2 cells	The different platelet derived growth factor isoforms act differentially on osteoblasts, the-AB isoform appearing the major responsible of the PRP chemiotaxis.
He et al. (2009) (43)	Beijing, China	Rat osteoblasts	PRF released autologous growth factors gradually and expressed stronger and more durable effect on proliferation and differentiation of rat osteoblasts than PRP *in vitro*.
Gassling et al. (2009) (44)	Kiel, Germany	Human osteoblasts, SaOS-2 cells	PRP application in cell cultures leads to higher levels of growth factors than PRF application.
Slapnicka et al. (2008) (45)	Brno, Czech Republic	Human osteoblasts	Activated PRP resulted in higher proliferation of osteoblasts compared with nonactivated PRP at concentrations of 10% and 25% in culture.
Goto et al. (2006) (46)	Kagoshima, Japan	MC3T3-E1 cells	PRP induces osteoblastic differentiation and mineralization of MC3T3-E1 cells.
Graziani et al. (2006) (47)	Pisa, Italy	Human osteoblasts	PRP preparations exert a dose-specific effect on osteoblasts. Optimal results were observed at a platelet concentration of 2.5.
Celotti et al. (2006) (48)	Milano, Italy	SaOS-2 cells	PRP dose-dependently stimulates both chemotaxis and cell proliferation.
Ogino et al. (2006) (49)	Kyushu, Japan	SaOS-2 cells	Cell proliferation was enhanced in all PRP groups in a dose-dependent manner.
Choi et al. (2005) (50)	Seoul, South Korea	Osteoblasts	Low PRP concentrations (1–5%) stimulated the viability and proliferation of cells.
Ferreira et al. (2005) (51)	Florianopolis, Brazil	Human osteoblasts	PRP promotes osteoblast proliferation.
Kanno et al. (2005) (52)	Fukuoka, Japan	SaOS-2 cells	PRP enhances human osteoblast-like cell proliferation and differentiation.
Graziani et al. (2005) (53)	Pisa, Italy	Osteoblasts	PRP has an enhancing effect on osteoblasts proliferation.
Okuda et al. (2003) (54)	Niigata, Japan	Osteoblast	PRP also stimulated DNA synthesis in osteoblast.

#### In Vivo Preclinical Studies

**Figure 3:**
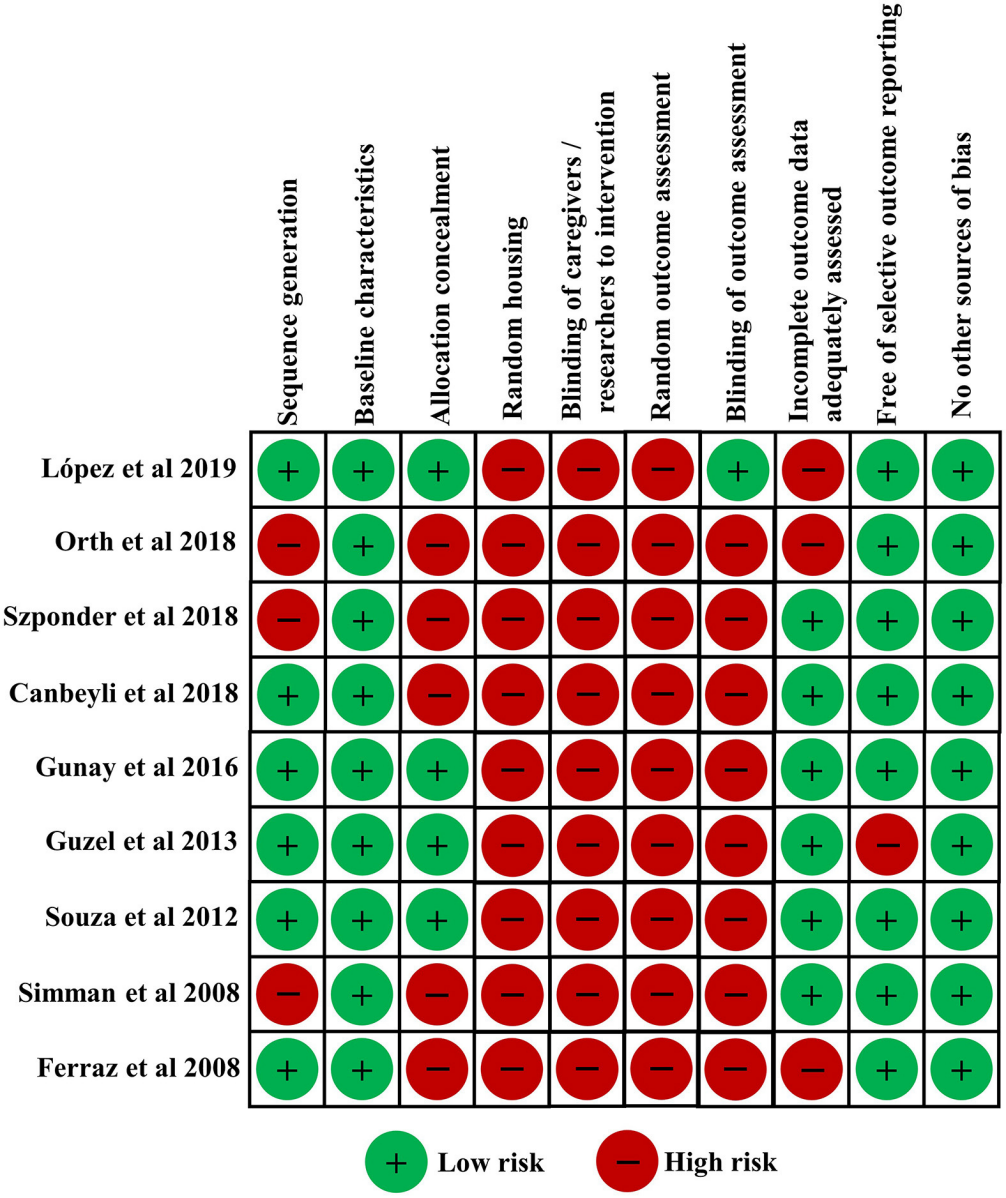
The risks of bias of all enrolled animal studies.

Nine *in vivo* preclinical studies (55–63) enrolled in this systematic review investigated the effect of PRP in the animal fracture model. The risk of bias of *in vivo* preclinical studies in this study was independently evaluated by two reviewers. All enrolled *in vivo* preclinical studies were considered to be of high quality. Risk of bias of all animal studies are shown in Figure 3. Among these studies, six studies carried out randomization. All enrolled animal studies reported a low risk of bias of baseline characteristics. Four studies conducted allocation concealment. Additionally, all enrolled animal studies reported a high risk of bias of random housing, blinding of researchers to intervention, and random outcome assessment. Eight studies reported a low risk of bias of free of selective outcome reporting. In addition, other obvious sources of bias in the animal studies were not detected.

The main details of all these animal studies are shown in Table 2. Among these animal studies, three studies were performed in Turkey, two in Brazil, each one in Spain, Germany, Poland, and USA. The type of animal model varied among the studies. The most used animal is rabbit (44%), followed by rat (22%), dog (22%), and mice (11%). Additionally, femur fracture (44%) is the most commonly used fracture model in all enrolled animal studies.

**Table 2:** The main details of animal studies.

**Author (Year)**	**Centre**	**Animal model**	**Types of bone**	**Treatments groups**		**PRP injection volume**	**Follow up**
				**PRP group**	**Control group:**		
López et al. (2019) (55)	Arucas, Spain	Dogs	Radius, ulna, tibia, fibula	External skeletal fixation and PRP injection	External skeletal fixation and saline solution injection	NR	6 months
Orth et al. (2018) (56)	Homburg, Germany	Mice	Femur	Internal fixation and PRP injection	internal fixation	0.2 μl	5 weeks
Szponder et al. (2018) (57)	Lublin, Poland	Rabbits	Tibia	Fixation surgery combined with β-tricalcium phosphate and PRP	Fixation surgery	NR	3 months
Canbeyli et al. (2018) (58)	Kirikkale, Turkey	Rabbits	Femur	K-wires fixation and PRP injection	K-wires fixation	NR	3 months
Gunay et al. (2016) (59)	Sanliurfa, Turkey	Rabbits	Rib	PRP injection	No treatment	3 ml	4 weeks
Guzel et al. (2013) (60)	Ordu, Turkey	Rats	Femur	K-wires fixation and PRP injection	K-wires fixation	0.2 ml	9 weeks
Souza et al. (2012) (61)	Araçatuba, Brazil	Dogs	Radius	External skeletal fixation and PRP injection	External skeletal fixation	1 ml	2 months
Simman et al. (2008) (62)	Ohio, USA	Rats	Femur	PRP injection	Saline injection	0.5 ml	4 weeks
Ferraz et al. (2008) (63)	Botucatu, Brazil	Rabbits	Orbit	BMP and PRP implant	BMP implant	0.3 ml	6 months

*NR, Not Reported*.

The most used treatment for bone fracture in animal fracture models is fixation surgery combined with PRP injection (67%), followed by PRP injection alone (22%), and the local administration of PRP plus BMP (11%). Six animal studies reported the volume of PRP injection, ranging from 0.2μl to 3ml. The follow-up duration of all animal studies ranged from 1 to 6 months.

All enrolled animal studies reported the procedures of PRP preparation, which are shown in Table 3. The PRP in animal studies are isolated from the respective animal models. Seven studies reported the blood volume used for PRP preparation, ranging from 1ml to 20ml. The most used centrifugation technique for PRP preparation is double centrifugation technique (67%), followed by single centrifugation technique (33%). Four studies reported PRP activation is calcium chloride while the second most used PRP activation was the combination of calcium chloride and bovine thrombin followed by bovine thrombin, and calcium gluconate. Three studies reported the platelet concentration in PRP, ranging from 2- to 4- fold over peripheral blood. Additionally, only one study tested the number of leukocytes in PRP.

**Table 3:** The PRP preparation and outcomes in animal studies.

**Author (Year)**	**PRP preparation**					**Outcomes**		
	**Blood volume**	**Centrifugation times**	**Activation**	**Platelet concentration**	**Leukocytes**	**Radiographic evaluation**	**Histopathological evaluation**	**Biomechanical evaluation**
López et al. (2019) (55)	20 ml	Two	Calcium chloride	2-fold over peripheral blood	Less than 0.2 × 10^6^/mL	The mean time for implant removal was shorter in PRP group.	NA	NA
Orth et al. (2018) (56)	NA	Two	NA	NA	NA	No differences in terms of bone volume.	An increased total callus area after two weeks and a reduced callus tissue area after five weeks in PRP group.	No significant differences in terms of polar moment of inertia.
Szponder et al. (2018) (57)	8.5 ml	Two	Bovine thrombin	NA	NA	Correct bone union was observed in the PRP group.	Immature fibrous bone tissue with clearly defined foci of angiogenesis were observed in PRP group.	NA
Canbeyli et al. (2018) (58)	5 ml	Two	Calcium chloride and bovine thrombin	Increase from 9.6–15.4 × 10^4^ to 22.9–48 × 10^4^ cells	NA	The mean radiological union score was higher in PRP group.	The cortical callus formation, woven bone area percentage, fibroblast proliferation, and mature bone formation were higher in PRP group.	NA
Gunay et al. (2016) (59)	8 ml	One	Calcium chloride	NA	NA	NA	The mean recovery plate thickness, fibrotic cell proliferation, capillary formation around the growth plate, callus formation were higher in PRP group.	NA
Guzel et al. (2013) (60)	1 ml	One	Calcium chloride	NA	NA	NA	Histological healing is better in PRP group.	Healing quantity and bone strength were better in PRP group.
Souza et al. (2012) (61)	8 ml	Two	Calcium chloride	Minimum increase of 338% from the basal platelet value.	NA	The radiographic healing score is higher in PRP group.	The histological evaluation is higher in PRP group.	NA
Simman et al. (2008) (62)	NA	Two	Calcium chloride and bovine thrombin	NA	NA	Callus to cortex width ratio were higher in the PRP group	Fracture histology showed enhanced bone formation in PRP group.	Three-point load bearing showed increased bone strength in PRP group.
Ferraz et al. (2008) (63)	5 ml	One	Calcium gluconate	NA	NA	No significant differences were found between two groups.	No significant differences were found between two groups.	NA

*NA, Not Available*.

The main outcomes *in vivo* preclinical studies are shown in Table 3. Seven studies conducted a radiographic evaluation of the PRP group and Control group. Of these seven preclinical studies, five studies (71%) reported positive and two studies (29%) reported negative radiographic outcomes. Eight studies conducted a histopathological evaluation of the PRP group and Control group. Of these eight studies, seven (88%) studies reported positive and one studies (13%) reported negative histopathological outcomes. Additionally, only three studies performed biomechanical tests of the PRP group and Control group. Of these three studies, two studies (67%) reported positive and one study (33%) reported negative biomechanical outcomes.

### Clinical Studies

**Figure 4:**
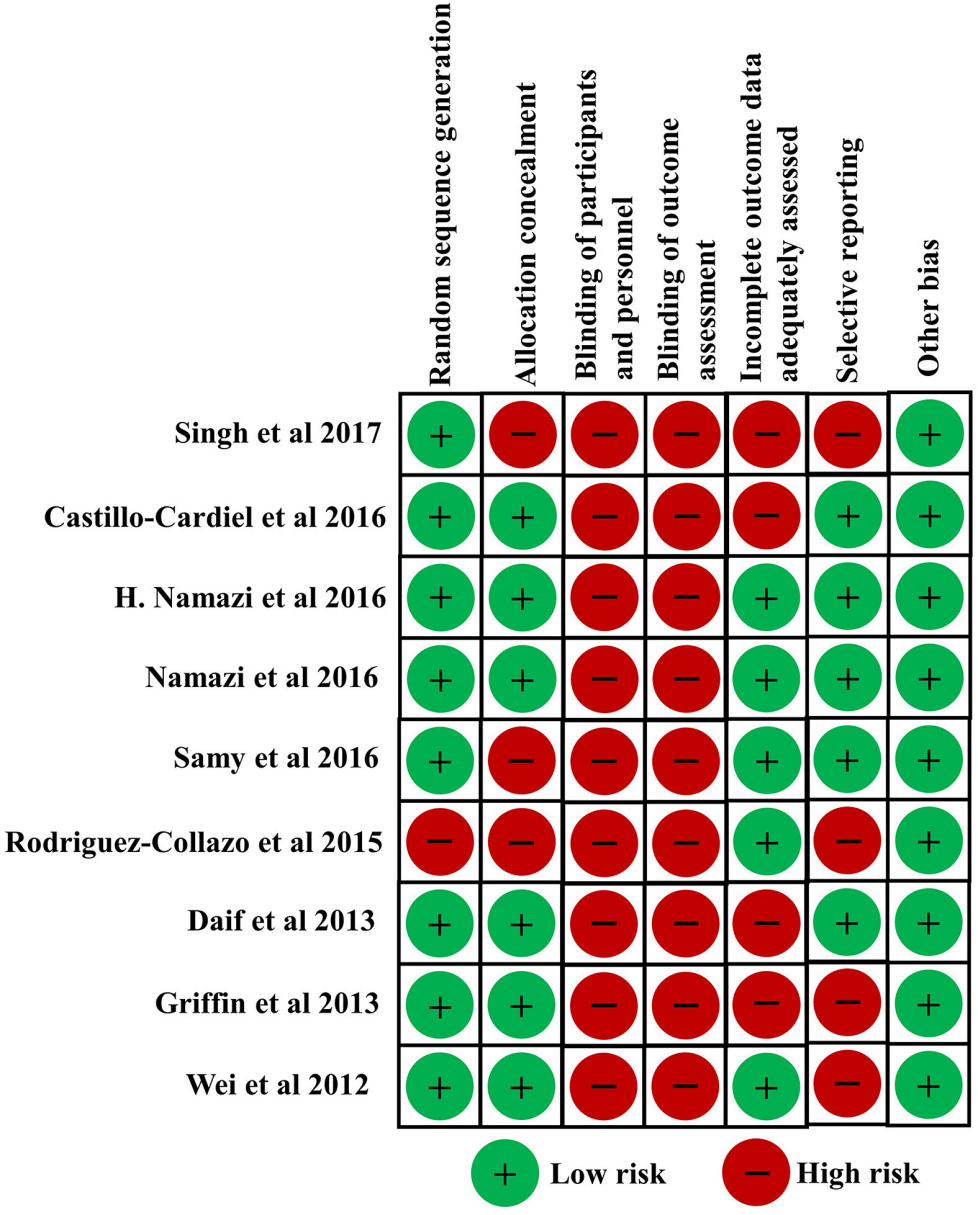
Risk of bias of all enrolled clinical studies.

Nine clinical studies (64–72) in this systematic review investigated the clinical effect of PRP for bone fracture treatment. The risk of bias of clinical studies in this study was independently evaluated by two reviewers according to the criteria in the Cochrane Collaboration for Systematic Reviews. All enrolled clinical studies were considered to be of high quality. Risk of bias of all enrolled clinical studies are shown in Figure 4. Among these studies, eight studies carried out randomization. Six studies conducted allocation concealment. None of the studies reported blinding of participants and personnel, or described blinding of outcome assessment. Five studies reported a low risk of bias of incomplete outcome data. In addition, other obvious sources of bias in the trials were not detected.

The demographic characteristics of clinical studies are shown in Table 4. Among all these enrolled studies, two studies were performed in Iran, two in Egypt, each one in USA, UK, Mexico, Indian, and China. The sample size of all these clinical studies ranged from 14 to 200. Eight studies reported the average age of patients, ranging from 30 to 83 years old. The most common fracture in these studies is femoral fracture (33%), followed by mandibular fracture (22%), radius fracture (11%), scaphoid fracture (11%), tibial and fibular fracture (11%), calcaneal fracture (11%). Only one study reported the smoking status of patients, and no significant differences of number of currently smoking patients were found between PRP group and control group (70). Among these clinical studies, eight studies applied local PRP injection combined with fixation surgeries in treatment for fracture patients, while only one study applied PRP injection alone as bone fracture treatment. Eight studies reported the volume of PRP injection, ranging from 1.5 to 14 ml. The follow-up period of all enrolled studies ranged from 3 to 72 months.

**Table 4:** The demographic characteristics of clinical studies.

**Author (Year)**	**Centre**	**Study design**	**The number of patients**	**Age (years)**	**Types of bone**	**Treatments**		**PRP injection volume**	**Follow up (months)**
						**PRP group**	**Control group**		
Singh et al. (2017) (64)	Haryana, India	Prospective	72	31.88	Femur	Intramedullary nailing and PRP injection	Intramedullary nailing	12–14 ml	6
Castillo-Cardiel et al. (2016) (65)	Guadalajara, Mexico	Prospective	20	31.6	Mandible	Internal fixation and PRP injection	Internal fixation	NR	3
H. Namazi et al. (2016) (66)	Shiraz, Iran	Prospective	30	32.87	Radius	Closed reduction, percutaneous pinning and PRP injection	Closed reduction, percutaneous pinning	3–5 ml	6
Namazi et al. (2016) (67)	Shiraz, Iran	Prospective	14	32.71	Scaphoid bone	PRP injection	Normal saline injection	1.5 ml	6
Samy et al. (2016) (68)	Tanta, Egypt	Prospective	60	30	Femur	Closed reduction, internal fixation with three cannulated screws, and PRP injection	Closed reduction, internal fixation with three cannulated screws	1.5ml	12–48
Rodriguez-Collazo et al. (2015) (69)	Chicago, USA	Retrospective	20	53.45	Tibia and fibula	PRP injection, cBMA, DBM in conjunction with the Ilizarov fixator	cBMA, DBM in conjunction with the Ilizarov fixator	3 ml	18
Daif et al. (2013) (71)	Cairo, Egypt	Prospective	26	32	Mandible	PRP injection, titanium miniplates and screws	Titanium miniplates and screws	5 ml	6
Griffin et al. (2013) (70)	Coventry, UK	Prospective	200	83	Femur	Cannulated screws and PRP injection	Cannulated screws	3–5 ml	12
Wei et al. (2012) (72)	Changsha, China	Prospective	175	NR	Calcaneus	Internal fixation, PRP injection, allograft;	Internal fixation and allograft	3–5 ml	72

*NR, Not Reported*.

All enrolled clinical studies reported the procedures of PRP preparation, which are shown in clinical studies is shown in Table 5. Eight studies (89%) reported the blood volume used for PRP preparation, ranging from 10 to 150ml. Seven studies reported centrifugation times during the procedure of PRP preparation, including five studies with double centrifugation technique and two studies with single centrifugation technique. Four studies used PRP activation, including one study with calcium gluconate, one study with calcium chloride, and two studies with calcium chloride and bovine thrombin. Only one study investigated the platelet concentration in PRP, which is 420% over peripheral blood. Additionally, none study investigated the number of leukocytes in PRP.

**Table 5:** The PRP preparation in clinical studies.

**Author (Year)**	**Blood volume**	**Centrifugation times**	**Activation**	**Platelet concentration**	**Leukocytes**
Singh et al. (2017) (34)	70ml	Two	Calcium gluconate	NA	NA
Castillo-Cardiel et al. (2016) (65)	20ml	One	Calcium chloride	NA	NA
H. Namazi et al. (2016) (66)	10ml	One	NA	NA	NA
Namazi et al. (2016) (67)	20ml	Two	NA	NA	NA
Samy et al. (2016) (68)	150ml	Two	NA	NA	NA
Rodriguez-Collazo et al. (2015) (69)	30ml	NA	NA	NA	NA
Daif et al. (2013) (71)	12ml	Two	Calcium chloride and bovine thrombin	NA	NA
Griffin et al. (2013) (70)	NA	NA	NA	NA	NA
Wei et al. (2012) (72)	100ml	Two	Calcium chloride and bovine thrombin	A platelet concentration of 420% was observed	NA

*NA, Not Available*.

The inclusion criteria, exclusion criteria, and main outcomes of fracture patients in enrolled clinical studies are shown in Table 6. Among these studies, four studies performed a radiographic evaluation of fracture patients. Singh et al. found that the intraoperative application of PRP led to a higher mean cortex to callus ratio when dealing with diaphyseal femur fracture (64). Castillo-Cardiel et al. (65) and Daif et al. (71) reported the local administration of PRP increased the bone density of mandibular patients. Additionally, Wei et al. reported that intraoperative application of PRP led to superior results of Bohler's Angle, the crucial angle of Gissane, and length, width, and height of the calcaneal body regarding radiographic assessment at 24 months and 72 months postoperatively (72). Five studies reported the bony union time of fracture patients. Also, in this case, results were controversial. Three studies (60%) reported positive and two studies (40%) reported negative results in terms of bony union time. Eight studies investigated the bone healing rate of fracture patients at the final follow up. Among these studies, seven studies (88%) reported no significant difference between the PRP group and Control group. Only one study (13%) showed superior results in the PRP group (68). Only two studies reported the information on revision surgery. Samy et al. reported that revision surgery was done for non-union cases with femoral neck fracture (68). Rodriguez-Collazo et al. reported that of the two patients in the PRP group who experienced delayed union, only one revision was required due to consistent pain (69). Four studies reported functional outcomes. Two studies reported that specific and usual activities scores were higher in PRP group (66, 67). Samy et al. reported that no significant differences were observed in terms of Harris hip score between PRP group and control group when dealing with femoral neck fracture (68). Wei et al. reported that no significant differences were observed in terms of AOFAS scores between PRP group and control group when dealing with displaced intra-articular calcaneal fracture (72). Three studies evaluated VAS scores of fracture patients. Two studies found that VAS scores were significantly lower in PRP group (66, 67). While Samy et al. reported that no significant differences were observed in terms of VAS scores between PRP group and control group when dealing with femoral neck fracture (68).

**Table 6:** The inclusion criteria, exclusion criteria, and main results of clinical studies.

**Author (Year)**	**Inclusion criteria**	**Exclusion criteria**	**Radiographic evaluation**	**Bony union time**	**Healing rate (PRP/Control)**	**Functional outcomes**	**VAS**	**Complication**
Singh et al. (2017) (64)	a. Age of 18 to 60 years. b. acute closed femoral shaft fracture (AO type 32).	Patients with open fracture, head injuries, pathological fracture, ipsilateral femoral fracture of proximal and distal segments, ipsilateral tibial fracture and fracture associated with bone disorders.	Mean cortex to callus ratio was high in PRP group.	No significant differences	100%/100%	NA	NA	NA
Castillo-Cardiel et al. (2016) (65)	Acute mandibular fracture	NR	Bone intensity and density were higher in PRP group	The bony union time is shorter in PRP group.	100%/100%	NA	NA	NA
H. Namazi et al. (2016) (66)	a. Age of 18 to 50 years. b. Simple intra-articular distal radius fracture (Frykman type 3, 4, 7, 8). c. less than 7 days.	a. Patients who refused to participate in research. b. Patients with previous joint destruction due to rheumatoid diseases. c. Previous intra-articular distal radius fracture, and limited range of motion of wrist due to malunion of previous fracture in this region. d. Joint collapse and step off in post-operation X-ray and the patient with subluxation of distal radioulnar joint in post-operation X-ray.	NA	NR	100%/100%	Specific and usual activities scores were higher in PRP group.	VAS was lower in PRP group.	NA
Namazi et al. (2016) (67)	Acute, unilateral nondisplaced middle-third scaphoid fracture type B2 according to Herbert classification	a. Patients who refused to participate in the study. b. Displaced scaphoid fracture, proximal pole fracture, fracture dislocations of the corpus or comminuted fracture (Herbert types B4 and B5). c. Presentation of > 7 days after injury, additional fracture of the wrist, previous wrist joint disease, and previous limited range of motion of the wrist joint.	NA.	No significant differences.	100%/85.71%	Specific and usual activities scores were higher in PRP group.	VAS was lower in PRP group	NA
Samy et al. (2016) (68)	a. Age of 20 to 45 years. b. Femoral neck fracture	a. Late presentation (more than 24 hours) after the fracture. b. Failure to achieve an acceptable reduction intraoperatively by closed methods. c. Pathological fracture. d. Auto-immune disease e.g., rheumatoid arthritis	NA	The bony union time is shorter in PRP group.	93.33%/83.33%	No significant difference in terms of Harriship score	No significant differences	NA
		and systemic lupus erythematosus. e. Endocrinal disorders. f. Need for bone graft						
Rodriguez-Collazo et al. (2015) (69)	Acute distal tibial and fibular fracture with a poor soft-tissue envelope.	NR	NA	The bony union time is shorter in PRP group.	80%/70%	NA	NA	No significant difference in terms of wound infection
Daif et al. (2013) (71)	Acute mandibular fracture	a. Any systemic diseases that may influence bone healing. b. presence of multiple or pathological fracture. c. Refusal of the patient to do surgical interference.	The bone density was higher in PRP group.	NR	100%/100%	NA	NA	NA
Griffin et al. (2013) (70)	a. Aged 65 years and above b. intracapsular hip fracture	Patients were excluded if they were managed non-operatively, presented late following their injury, had serious injuries to either lower limb that interfered with rehabilitation of the hip fracture, or had extant local disease precluding fixation, for example, local tumor deposit and symptomatic ipsilateral hip osteoarthrosis.	NA	NR	97.56%/98.72%	NA	NA	No significant difference in terms of wound infection
Wei et al. (2012) (72)	Displaced intra-articular calcaneal fracture (Sanders type III).	Any evidence of nerve or blood vessel injury.	The Bohler's Angle, the crucial angle of Gissane, the length, and height of the calcaneal body were higher in PRP	NR	100%/100%	No significant differences in terms of (AOFAS) ankle-hind-foot scoring	NA	Six patients in control group developed wound infection

*NR, Not Reported; NA, Not Available*.

Among these studies, only three studies reported postoperative wound infection, which is a type of fracture-related infection. Griffin et al. (70) and Rodriguez-Collazo et al. (69) reported no significant differences were found between the PRP group and Control group. Rodriguez-Collazo et al. reported that the two patients with wound infection were treated with oral antibiotics. Wei et al. (72) reported that the rate of postoperative wound infection in the allograft + PRP group was significantly lower than that of the allograft-only group when dealing with displaced intra-articular calcaneal fracture. Wei et al. reported that six cases of infection in the allograft were treated by sustained suction with negative pressure for a week, and the incision was closed using secondary suturing.

## Discussion

As there was no related systematic review published yet, the goal of this overview was to systematically review all available preclinical and clinical studies concerning PRP for bone fracture treatment. The present review confirmed the continuing interest and debate about PRP as an additional treatment for bone fracture. All enrolled studies in this systematic review were published between 2003 and 2020. Among these studies, most of the enrolled studies are preclinical, and clinical researches account for only a small part. Additionally, we found that the most commonly used fracture model *in vivo* was femur fracture. In our opinion, current enrolled studies are representative of the tendency for application of PRP for bone fracture treatment.

The healing process of bone fracture is complex and involves a well-orchestrated series of biological events initiated by many growth factors *in vivo* (73–75). Deriving from peripheral blood, PRP can release considerable amounts of growth factors, such as fibroblast growth factor, platelet-derived growth factor, transforming growth factor, vascular endothelial growth factor, insulin-like growth factor, which can activate related intracellular and extracellular molecular-signaling pathways to enhance bone regeneration (76, 77). Theoretically, the rationale behind PRP use in bone fracture treatment is that PRP constitutes a high concentration of autologous growth factors that are critical to regulate the tissue healing process, which is quite similar in all kinds of tissues (78). The main intent of *in vitro* preclinical studies in present systematic review is to investigate the effect of PRP on osteoblast-like cells, and the analysis results have highlighted the positive effects an overall positive effect of PRP on osteoblast-like cells. Most of *in vitro* preclinical studies supported the role of PRP in the adhesion, migration, and proliferation of osteoblast-like cells. Additionally, some studies reported PRP stimulated osteoblast activity and cytokine/chemokine release. Besides some controversial results, most of *in vitro* preclinical studies in this systematic review induced osteoblastic differentiation. The platelet concentrations in PRP exert a dose-specific effect on osteoblasts. Meanwhile, cell viability and migration assay have demonstrated a detrimental effect at high platelet concentration (31). The positive effects of PRP on osteoblast-like cells provide convincing evidence for the clinical application of PRP as a potent tool to facilitate bone regeneration. Many kinds of cells, including osteoblasts, osteoclasts, and endothelial cells, take part in the different phases of the bone healing process (79–81). Previous studies are mainly about the effect of PRP on osteoblasts, but there are still few studies concerning the effect of PRP on osteoclasts. Vascularization is another important part of bone healing, the angiogenesis effect of PRP has been confirmed by many researches (82–84). However, there are still few studies reporting the angiogenesis effect of PRP in the bone healing process.

For the further clinical application of PRP, as an important part of preclinical research, animal research plays a vital role in effective prediction of PRP administration *in vivo*. Besides some controversial results, the systematic analysis of animal studies published up to now shows an overall positive effect of PRP in bone fracture treatment in terms of radiographic, histopathological, and biomechanical evaluation, which provide the theoretical basis for the clinical application of PRP in bone fracture. More than half of animal studies performed fixation surgery combined with intraoperative administration of PRP. Most of animal fracture models in this systematic review are small animals like mice, rats, and rabbits, which have advantages of low-cost, easy handling, and short period of bone healing. However, compared to large size animal models, small animals are less reliable in imitating bone structure and anatomy of humans (85). Interestingly, all the enrolled studies using the dog model showed positive results using PRP in bone fracture treatment. In contrast, the controversial results in animal studies mainly come from small animal studies.

The main aim of clinical studies was to investigate the effects of PRP in fracture patients. Most of the enrolled clinical studies performed intraoperative administration of PRP as an additional approach for bone fracture treatment. The systematic analysis of clinical studies shows an overall positive effect of PRP in radiographic evaluation. More than half of the clinical studies reported that PRP shortened bony healing duration. In our opinion, for fracture patients, especially in the elderly, shortening the bone healing time could shorten the bedridden time and immobilization and result in decreasing the incidence of pulmonary infection, thrombosis, and bedsore. Furthermore, the systematic analysis of clinical studies found PRP could not improve the healing rate, which might be associated with a high healing rate of closed fractures. However, the results of functional outcomes and VAS are controversial. Interestingly, all enrolled studies concerning wrist fractures showed that PRP could relieve pain and improve the functional outcomes in patients with wrist fractures. Previous studies found that PRP contains a high level of IL-1 receptor antagonist, which could inhibit IL-1 and result in decreasing the amount of substance P, a significant pain transmitter (86, 87). Also, the high level of hepatocyte growth factor (HGF) in PRP could also mediate the signal of NF-kb, resulting in decreasing the level of Cox-1, Cox-2, PGE2 (88). The incidence of postoperative complications is an important index to evaluate the clinical safety of PRP. Currently, no severe complications were reported in all enrolled studies.

All enrolled animal and clinical studies reported the procedure of PRP preparation. The most commonly used PRP activator in animal and clinical studies is calcium chloride. More than half of animal and clinical studies performed two centrifugation times in PRP preparation. Although the easy procedure of preparation and the promising results make PRP a potential therapeutic method to promote bone healing, the PRP preparation procedures of animal studies and clinical studies are heterogeneous. Many different methods of PRP preparation were reported in enrolled animal and clinical studies, leading to a difficult interpretation of PRP regenerative properties in the process of bone healing. Additionally, the effect of different centrifugation times and different activators on the osteogenic ability of PRP is still unclear. Additionally, leukocytes have many important roles in process of tissue healing, and their inclusion in PRP results in increased platelet concentrations (89–91). Generally, the levels of PDGF and TGF-β1 were higher in preparations that contain leukocytes compared to leukocyte-poor PRP (92). Leukocytes could not only secrete many growth factors, such as PDGF, VEGF, TGF-β1, and IGF, but also express many proteinases, including serine and metalloproteinases (93, 94). Consequently, leukocyte-rich PRP could attract other leukocytes, prevent infection, and enhance platelet production by megakaryocytes (95). However, some researchers found that leukocytes damaged surrounding tissues by excessive release of reactive oxygen species, which results in diminishing PRP efficacy in the process of tissue healing (89, 96). However, there are few studies focusing on the differences between leukocyte-rich PRP and leukocyte-poor PRP when dealing with bone fracture. In our opinion, further researches are still needed to investigate the role of leukocytes in PRP for the bone healing process.

There are several limitations to this systematic review. Firstly, due to the high heterogeneity of the enrolled animal and clinical studies in the present review, a meta-analysis of the main results was not performed. Secondly, there are many different protocols of PRP use in enrolled studies and lack of standardization in PRP preparation procedures. Long-term consensus on the standardization of PRP for bone fracture treatments still needs further large-scale trials. Additionally, the characteristics of PRP in enrolled studies, such as platelet concentration, the numbers of leukocytes, were not discussed in detail in the enrolled studies. Thirdly, PRP might also be a promising approach in the treatment of patients with a pathological fracture or periprosthetic fracture. However, all enrolled clinical studies in this systematic review were performed in the patients with traumatic fracture.

## Conclusion

The present systematic review confirmed the continuing interests of PRP as an additional treatment for bone fracture. Preclinical studies highlighted the potential value of PRP as promising therapy for bone fracture. However, the preclinical evidence did not translate into a similar result in the clinical studies. PRP can shorten fracture healing time, but it cannot improve fracture healing rate in fracture patients. Meanwhile, characteristics of PRP, such as platelet concentration, the numbers of leukocytes, still need further researches. Although the present systematic review could not fully prove the role of PRP in bone healing, the easy procedure of preparation and the promising results make PRP a potential therapeutic method for bone fracture treatment.

## Data Availability Statement

The original contributions presented in the study are included in the article/supplementary material, further inquiries can be directed to the corresponding author/s.

## Author Contributions

XD: conceptualization and supervision. FX and YZ: methodology, resources, and data extraction. RL: software. YZ and FX: validation. FX and RL: formal analysis. FX: investigation. YZ: writing—original draft preparation, writing—review, and editing. All authors have read and agreed to the published version of the manuscript.

## Conflict of Interest

The authors declare that the research was conducted in the absence of any commercial or financial relationships that could be construed as a potential conflict of interest.

## Publisher's Note

All claims expressed in this article are solely those of the authors and do not necessarily represent those of their affiliated organizations, or those of the publisher, the editors and the reviewers. Any product that may be evaluated in this article, or claim that may be made by its manufacturer, is not guaranteed or endorsed by the publisher.
